# Freshwater wetlands: fertile grounds for the invasive *Phragmites australis* in a climate change context

**DOI:** 10.1002/ece3.1576

**Published:** 2015-07-24

**Authors:** Marie-Andrée Tougas-Tellier, Jean Morin, Daniel Hatin, Claude Lavoie

**Affiliations:** 1 École supérieure d'aménagement du territoire et de développement régional and Centre de la science de la biodiversité du Québec, Université LavalQuébec City, Québec, Canada; 2 Service météorologique du Canada, Section Hydrologie et Écohydraulique, Environnement CanadaQuébec City, Québec, Canada; 3 Ministère des Forêts, de la Faune et des Parcs du Québec, Direction de la gestion de la faune de l'Estriea de Montréal, de la Montérégie et de LavalLongueuil, Québec, Canada

**Keywords:** Climate change, common reed, *Phragmites australis*, St. Lawrence River, water level, wetland

## Abstract

Climate change will likely affect flooding regimes, which have a large influence on the functioning of freshwater riparian wetlands. Low water levels predicted for several fluvial systems make wetlands especially vulnerable to the spread of invaders, such as the common reed (*Phragmites australis*), one of the most invasive species in North America. We developed a model to map the distribution of potential germination grounds of the common reed in freshwater wetlands of the St. Lawrence River (Québec, Canada) under current climate conditions and used this model to predict their future distribution under two climate change scenarios simulated for 2050. We gathered historical and recent (remote sensing) data on the distribution of common reed stands for model calibration and validation purposes, then determined the parameters controlling the species establishment by seed. A two-dimensional model and the identified parameters were used to simulate the current (2010) and future (2050) distribution of germination grounds. Common reed stands are not widespread along the St. Lawrence River (212 ha), but our model suggests that current climate conditions are already conducive to considerable further expansion (>16,000 ha). Climate change may also exacerbate the expansion, particularly if river water levels drop, which will expose large bare areas propitious to seed germination. This phenomenon may be particularly important in one sector of the river, where existing common reed stands could increase their areas by a factor of 100, potentially creating the most extensive reedbed complex in North America. After colonizing salt and brackishwater marshes, the common reed could considerably expand into the freshwater marshes of North America which cover several million hectares. The effects of common reed expansion on biodiversity are difficult to predict, but likely to be highly deleterious given the competitiveness of the invader and the biological richness of freshwater wetlands.

## Introduction

Climate change and the introduction of invasive species are major threats to biodiversity. The synergy of these two drivers of change will probably affect some ecosystems more than others, such as wetlands, where fluctuating water levels have a high influence on the composition and distribution of plant assemblages (van der Valk et al. 1994; Casanova and Brock 2000; Hudon 2004; Webb et al. 2012). Climate warming over the next few decades will likely affect the timing, distribution, frequency, and duration of floods. This will especially be true for the St. Lawrence–Great Lakes hydrographic system, one of the most important waterways in North America. The water supply from the Great Lakes could significantly drop in the near future because of high evaporation levels during winter due to a lack of extended ice cover (Croley 2003). Water levels of the St. Lawrence River could consequently fall by as much as 80–100 cm (Croley 2003; Fagherazzi et al. 2005; Morin et al. 2009). A change of this magnitude to the hydrologic regime could foster the spread of several invasive wetland plants following the exposure of large germination grounds (Hudon 2004).

The common reed (*Phragmites australis* (Cav.) Trin. ex Steud.), considered to be the most invasive plant in the marshes of northeastern North America (Lelong et al. 2007; Tulbure and Johnston 2010; Kettenring et al. 2012), will likely flourish under the new conditions created by climate change. The exotic haplotype M (Saltonstall et al. 2004) was introduced along the St. Lawrence River approximately 100 years ago (Lelong et al. 2007). Populations were fairly rare until the 1960s and 1970s, and were primarily found along the river. Since the 1980s, they have spread inland in Québec and Ontario (Canada) as a result of the road network development, as adjacent drainage ditches provide very good habitat for the plant (Lelong et al. 2007; Jodoin et al. 2008). Although reedbeds do not cover very large areas in the St. Lawrence River wetlands, recent surveys indicated that the situation is rapidly changing: in some marshes, the invaded area has more than doubled since the beginning of the 2000s (G. Létourneau, unpubl. data).

Several studies have shown that in freshwater wetlands, prolonged periods of soil surface exposure foster the establishment of new common reed populations and their subsequent spread through vegetative propagation (Hudon et al. 2005; Whyte et al. 2008; Tulbure and Johnston 2010; Wilcox 2012). These new populations are essentially created through the germination of a few seeds (Belzile et al. 2010; McCormick et al. 2010; Kettenring et al. 2011; Kirk et al. 2011; Albert et al. 2015). Seed germination occurs on wet (nonflooded) soils devoid of vegetation (Weisner and Ekstam 1993; Coops and Van der Velde 1995; Tulbure and Johnston 2010). Reedbeds generally form dense patches with stems reaching approximately 4 m in height (Gagnon Lupien et al. 2015). The yearly biomass produced by the plant results in a thick layer of litter that decomposes slowly (van der Valk et al. 1991; Warren et al. 2001; Windham 2001). The accumulation of organic matter alters sediment dynamics, creating a build-up of vegetation that reduces water flow, changes topography, and plays a role in the gradual drying of invaded marshes (Windham and Lathrop 1999; Rooth et al. 2003).

Whereas the negative effects of a common reed invasion on native flora are tangible and well documented (Keller 2000; Meyerson et al. 2000; Lavoie et al. 2003), its impacts on fish and wildlife are not as clear. Although faunal assemblages in brackishwater marshes are significantly affected by the presence of the common reed (Dibble et al. 2013), freshwater fish (Larochelle et al. 2015), amphibian (Mazerolle et al. 2014), and bird assemblages (Meyer et al. 2010; Gagnon Lupien et al. 2015) do not appear to be strongly disturbed by the invader. It is nevertheless possible that the number and extent of common reed populations in freshwater marshes have not yet reached a threshold beyond which adverse effects on biodiversity become more important (Gagnon Lupien et al. 2015). This hypothesis remains to be tested, but a model predicting the distribution of the common reed under climate change scenarios would be extremely useful for evaluating the risk to wetland biodiversity.

The specific objectives of this study were to develop a model enabling us to map the distribution of potential germination grounds for the common reed in the St. Lawrence River wetlands under current climate conditions and then use this model to predict their future distribution under two climate change scenarios simulated for 2050. We hypothesized a significant increase in the area that could potentially be occupied by common reed stands in 2050, particularly in Lake St. Pierre, a shallow widening of the river with more than 16,000 ha of wetlands, in which common reed stands are, for the moment, relatively uncommon.

## Materials and Methods

### Study area

**Figure 1:**
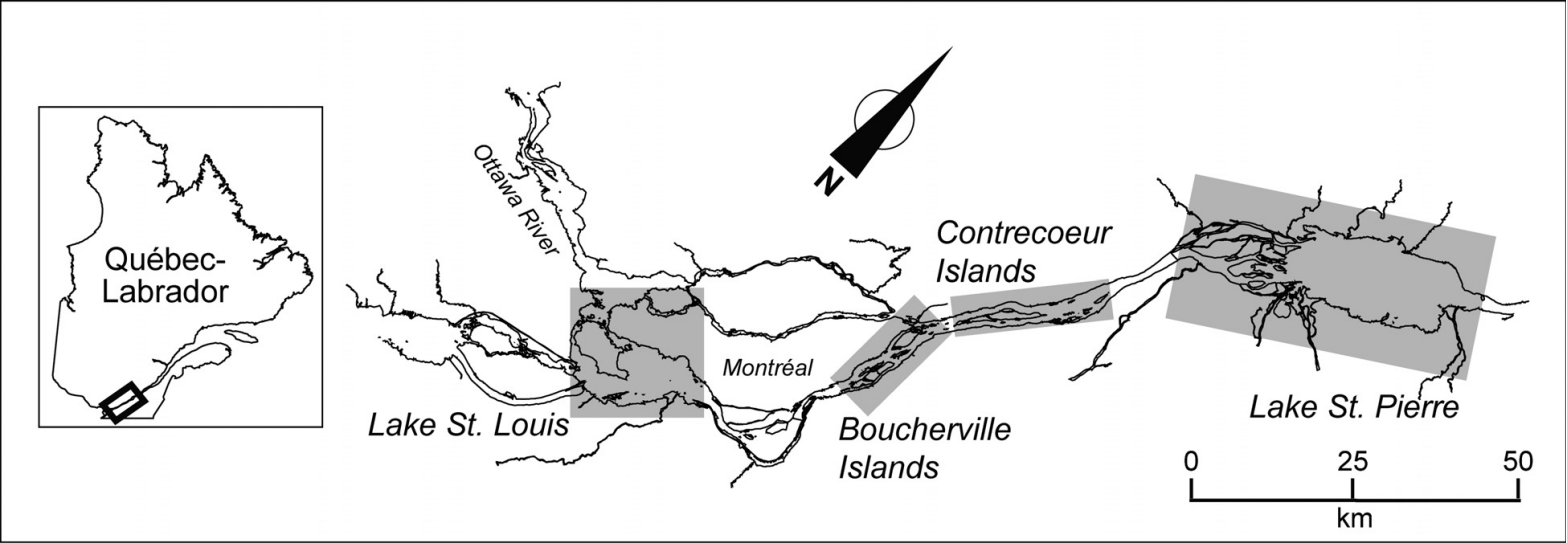
Map of the study area (St. Lawrence River, Québec, Canada). The four sectors of the river under study are delineated by gray boxes.

The impact of climate change on the spread of the common reed was assessed for the St. Lawrence River wetlands, in a section of the river 150 km long (Fig. 1), from Lake St. Louis (45°19′N; 73°56′W) to Lake St. Pierre (46°16′N; 72°38′W). The St. Lawrence River flow is fed by the Great Lakes and Ottawa River watersheds, which drain an area of 917,000 km^2^. Variations in river flow can largely be attributed to fluctuations in precipitation. However, the Moses-Saunders Dam (Cornwall, Ontario) regulates the flow to minimize extreme fluctuations in water level, both along the Great Lakes and the river. Spring floods normally increase water flow in Lake St. Pierre to 13,000 m^3^sec^−1^ in April, while it might only reach 9000 m^3^sec^−1^ during the low flow periods in August and January. The vast majority of wetlands (nearly 90%) are located in the Lake St. Pierre sector (Jean and Létourneau 2011). In the other sectors (Lake St. Louis, Boucherville Islands, Contrecœur Islands), wetlands cover smaller areas and are mainly found near islands. Overall, wetlands of the St. Lawrence River are made up by high marshes (55% of the total surface area), low marshes (26%), and swamps (19%; Létourneau and Jean 1996).

### Modeling approach

The basic assumption of our common reed model is that a new reed stand arises from seed. This assumption is now strongly supported – especially for Québec – by several recent field and genetic studies (Brisson et al. 2008; Belzile et al. 2010; McCormick et al. 2010; Kettenring et al. 2011; Kirk et al. 2011; Albert et al. 2015). Consequently, the model took into account the parameters (climatic, hydrologic, and biotic conditions) associated with common reed sexual reproduction and seedling establishment. Subsequent vegetative propagation was not addressed in this study. Competition with other plant species slows the vegetative propagation of the common reed, but it does not prevent its spread (Bellavance and Brisson 2010). In other words, the model focussed on locating suitable germination grounds in wetlands as starting points for the establishment of new common reed stands. Several steps were required to develop this model; these steps are presented in the following paragraphs.

#### Step 1: Distribution of common reed stands

Data on the historical distribution of common reed stands were gathered through aerial photographs. It is possible to distinguish stands of a certain size (≥5 m^2^) in black and white or infrared photographs at a scale ≤1:15,000 (Warren et al. 2001; Wilcox et al. 2003; Hudon et al. 2005; Maheu-Giroux and de Blois 2005). This work was performed for the only sector in which common reed stands were both abundant and old enough to build a valid historical reconstruction, that is, the Boucherville Islands. The database used is based on a mapping carried out by Hudon et al. (2005), which covered 1980 (1 ha of common reed stands) to 2002 (33 ha). Using a geographic information system, the centroid of each large common reed stand, which was primarily circular in shape, was georeferenced when first detected. The most recent genetic data gathered at Boucherville Islands (Albert et al. 2015) indicated that each stand was monoclonal, created through the germination of a single seed, followed by the extension of rhizomes and stolons in every direction. The centroid of a common reed stand therefore roughly corresponds to the location of the germination bed that permitted successful reed stand establishment.

Current distribution of common reed stands was mapped through remote sensing using high-definition WorldView–02 and QuickBird–02 satellite images taken between August 15 and September 18, 2010. A configuration of four multispectral bands (near infrared, red, green and blue) was used, providing a spatial resolution of approximately 2.4 m. Common reed stands were identified and delineated using spectral signatures developed by Létourneau and Jean (2005) based on training areas located in the St. Lawrence River wetlands. Because the spectral signature of the common reed is somewhat similar to that of the reed canary grass (*Phalaris arundinacea* L.), a plant species also commonly found in the St. Lawrence River floodplain, the map created from satellite images was validated in the field from helicopter (October 18, 2011) and by foot (September 13 and 14, 2011). In all, 240 previously mapped common reed stands were visited for validation, primarily in the Lake St. Louis, Boucherville Islands, and Lake St. Pierre sectors. These stands were selected through a stratified random sampling, which enabled us to confirm the presence of the common reed based on a range of stand areas (0–10 m^2^, 11–50 m^2^, ≥51 m^2^). The map was then adjusted based on results obtained during validation. The centroid of each common reed stand retained on the validated map was localized using the same method used for the historical distribution data.

#### Step 2: Parameters conducive to common reed establishment

One of the challenges in modeling common reed establishment in a natural environment resides in the fact that it is extremely difficult to determine the exact moment of seed germination, because there is always a lag of a few years between seed germination and the formation of a reed stand (of about 5 m^2^) that can be detected on aerial photographs or satellite images. It was thus impossible to characterize conditions conducive to seed germination and seedling establishment based on historical data. As a result, the model could only be developed using a deductive approach (Ottaviani et al. 2004) and was essentially based on the most current knowledge regarding the biology of the common reed.

Bare, wet (not flooded) soils are necessary for a new common reed stand to develop from a seed (Galinato and van der Valk 1986; Coops and Van der Velde 1995; Hudon et al. 2005). Such exposure conditions are present along the St. Lawrence River during summer-long episodes of low water levels or in areas with anthropogenic disturbance (Hudon 1997, 2004; Hudon et al. 2005). These nonflooded conditions must be present at the beginning of the growing season. Seedlings need at least 120 days of growth; otherwise, they are not able to accumulate sufficient reserves, which will hamper their survival the following year, particularly during spring floods and frosts (Haslam 1971; Weisner and Ekstam 1993; Ekstam and Forseby 1999). Furthermore, common reed seed germination occurs at a mean daily air temperature of ≥10°C (Haslam 1972; Ekstam and Forseby 1999) and a daily temperature range of ≥10°C (Ekstam and Forseby 1999). However, very early germination is not necessarily beneficial because seedling risk exposure to late spring frosts (Weisner and Ekstam 1993). We thus considered that germination and subsequent seedling growth periods could not start until the first day of the year when mean daily temperatures reached 10°C (and were not followed by temperatures below 0°C) until the first autumn frost.

Light conditions also influence common reed germination and seedling survival (Haslam 1972), and a dense tree cover can strongly reduce seedling establishment and growth (Mal and Narine 2004; Lelong et al. 2009; Albert et al. 2013). Therefore, a robust common reed model must also consider the various classes of wetlands that can be present in a study area, each with their particular tree cover. For this study, we used the ten classes defined specifically for the St. Lawrence River by Desgranges et al. (2006), but only the three classes associated with the presence of trees (open treed swamp, closed treed swamp, and damp forest) were considered obstacles to common reed germination and seedling survival (no establishment).

#### Step 3: Structure of the common reed model

The common reed model was developed within the infrastructure of the *Integrated Ecosystem Response Model* (IERM), a high-resolution spatial database developed by Environment Canada to evaluate the impact of water level fluctuations and flow regulation on the St. Lawrence River ecosystems (Morin et al. 2005; Morin and Champoux 2006). IERM has been successfully used to predict the impact of water level changes on fish and bird reproduction, and on wetland vegetation (Desgranges et al. 2006; Morin et al. 2006; Mingelbier et al. 2008). The methodology used to build the basic datasets and to run IERM is described in Morin and Champoux (2006) and Morin et al. (2006). In summary, IERM is based on a spatial grid whose nodes are 20–80 m apart in shallow water areas and 160 m apart in the navigation channel. Several information layers measured in the field or modeled (topography, bathymetry, substratum, water level, current velocity, aquatic vegetation, emergent vegetation) are associated with each node. IERM not only models fluvial physics, but also simulates the spatiotemporal development of ten wetland vegetation classes (Desgranges et al. 2006; Morin et al. 2006).

The location of a common reed stand did not necessarily correspond to the exact location of a node of the IERM grid. The IERM node network was therefore transformed into a square polygon grid with surface areas varying from 0.04 to 2.56 ha, depending on the distance between nodes in the original grid. The information associated with the central node of each polygon was then applied to the entire polygon. The presence of at least one common reed stand in a polygon was enough to consider this polygon as conducive to the establishment of the invader. The spatially explicit information layers used in this study were topography, water level, and wetland class. Air temperature data, available at only one station, were extracted from the historical series measured at the Montréal Airport (Dorval-Trudeau; Environment Canada, 2013). This series is the longest (1953–2012) and most complete available in the study area. These values were also used as references for the entire area (Morin et al. 2005). Temperature data were thus not spatially distributed: they were associated with each node (polygon), but were the same for all the nodes of the modeling domain. The other data, measured or simulated for each node (polygon) of the grid, were extracted from the models developed by Morin et al. (2005).

The parameters conducive to common reed seed germination and seedling survival, as presented above (step 2), were integrated into IERM in the form of an algorithm. This algorithm was used to query the geodatabase on a daily basis (for a given year), which then returned all sites whose attributes were within the required parameters, thus identifying potential common reed germination grounds. For example, to verify the presence of an appropriate water level, which required that a site not be flooded at the beginning of the growing season, the water level was examined (based on IERM data) for all polygons each day of a given year. A count began as soon as all conditions conducive to common reed seed germination and seedling survival were met in a polygon. If there were nonflooded conditions, the other parameters conducive to germination were met, and the seedlings had at least 120 days of appropriate growing conditions (no frost), the polygon was identified as a potential site for common reed establishment. Data on temperature and water level measurements were available on daily means, while the wetland classes were reassessed yearly based on environmental conditions observed the previous years. Data related to land use and topography were kept constant.

A daily time step was used for the historical series analysis. However, a quarter-monthly time step was used for the climate change simulations, because the water level series for the St. Lawrence River were available only in quarter-monthly means. The quarter-monthly time step divides time into 7- or 8-day blocks, for a total of 48 quarter-monthly time steps per year. Using quarter-monthly time steps permits the comparison of individual quarter-monthly time steps across years and has the advantage of not being influenced by the number of days in a year (Morin et al. 2009).

#### Step 4: Calibration of the common reed model

The invasion stage, which reflects the proportion of invaded to invadable areas, directly impacts the ability of a model to predict areas vulnerable to invasion (Václavík and Meentemeyer 2012). If the invasion is advanced, one can presume that the species occupies most of the sites that foster its establishment; it is then easy to establish a correlation between site conditions and their likelihood of being invaded. It is much more difficult to establish such a correlation if the invasion is still in the early stages: the invader absence at a given site does not necessarily correspond to unfavorable local conditions. Thus, the accuracy of the parameters associated with common reed germination and seedling survival was verified in the Boucherville Islands, that is, in the sector of the river containing one of the oldest populations of the plant, the greatest surface area of common reed stands, and a well-documented invasion history. To do so, the common reed distribution, as simulated by IERM, was compared to the real distribution (see step 1). The simulated distribution was generated from 1980 (beginning of the common reed invasion in this sector) to 2009 and took into account the historical environmental data integrated into IERM. Given that satellite images were taken during the 2010 growing season, the period used for modeling ended in 2009.

The comparison was made using a confusion matrix (Fielding and Bell 1997), which helped to determine (1) the number of polygons with successful common reed seedling establishment and that were also identified by IERM as potential establishment sites between 1980 and 2009 (true positives); (2) the number of polygons with seedling establishment but that were not identified as such by IERM (false negatives); (3) the number of polygons with no seedling establishment but where the common reed should have established stands according to IERM (false positives); and (4) the number of polygons with no seedling establishment and that were also identified by IERM as not being conducive to the emergence and survival of common reed seedlings (true negatives). To improve the model performance, various combinations of parameters were tested, for example, the inclusion or exclusion of the average daily temperature, daily temperature amplitude, water level, and wetland class. Once calibrated, the common reed model was applied to the entire study area.

Given that the common reed invasion in the Boucherville Islands is relatively recent and still ongoing, we hypothesized that the polygons identified as false positives, while having a high germination potential, had not yet been invaded either as a result of insufficient time or the random dispersal of seeds. It was also possible that stand size within a false-positive polygon was simply too small to be detected via satellite imagery. To confirm this hypothesis, 213 of the 317 false-positive polygons (randomly selected) in the Boucherville Islands sector were visited between October 1 and 5, 2012. Each polygon was verified by an observer on foot. Searching continued until either common reed was found or the entire polygon had been covered. If common reed stems were found, their origin (from seed – not connected by rhizomes or stolons to adjacent stands – or from vegetative propagation) was noted.

#### Step 5: Performance assessment of the common reed model

The common reed model performance was assessed using indices derived from the confusion matrix (Fielding and Bell 1997). The sensitivity index indicates the proportion of polygons in which seedling establishment occurred, and which were identified by the model as potential germination grounds, in relation to all existing common reed polygons. The specificity measures the proportion of polygons identified by the model as unfavorable to seedling establishment among the entire collection of polygons, and where seedlings did not establish. Lastly, the correct classification rate measures the performance of the entire model. This rate is the total proportion of polygons where concordance exists between the model and reality. Model performance was assessed for the entire study area and for each of the four study sectors.

#### Step 6: Future distribution of the common reed and climate change scenarios

Once the model performance assessed, it was used to identify sites favorable to the establishment of common reed through seeds, either based on current or simulated climatic conditions associated with various climate change scenarios. For this study, the periods compared were 1961–1989 (reference period with actual climate data) and 2040–2069 (simulated climate data; Croley 2003; Fagherazzi et al. 2005; Morin et al. 2009). Simulations of river flows and air temperatures modified by climate change were carried out using mean values from the 2040–2069 period, commonly associated with the year 2050. The simulated climate data were extracted from two general circulation models integrating greenhouse gas emission scenarios, one resulting in very warm and wet conditions (HadCM3 A1FI) and the other under very warm and dry conditions (CGCM2 A21; Mortsch et al. 2005). Among all the climate change scenarios available for modeling (Mortsch et al. 2005), these two scenarios were the most recent ones providing hydrologic series for the study area and corresponded to conditions that differed most from those currently known for the St. Lawrence River sector under study. The hydrologic series with climate change data were only available in a quarter-monthly time step format.

Each climate change scenario was associated with monthly temperature differences, also known as monthly deltas, between current values and those of the 2040–2069 period. From the overall number of climate models developed for small-scale gridding (approximately 400-km^2^ grids; Mortsch et al. 2005), a unique monthly value was applied to the entire study area. Daily temperature data recorded at Dorval-Trudeau (1961–1989) were used as reference. The monthly temperature deltas were then applied to each quarter-monthly time step of the series measured at Dorval-Trudeau for the entire period studied. The monthly differences applied to the reference temperature data for scenario HadCM3 A1FI were, on average, 4°C, and ranged from 3.2°C (May) to 5.2°C (August). For scenario CGCM2 A21, the average monthly temperature difference was 3.0°C and ranged from 1.0°C (December) to 5.9°C (January). The beginning and the end of the common reed germination and seedling growth period, determined using mean daily temperature values, were recalculated for each climate change scenario based on the adjusted temperature data available in a quarter-monthly time step format.

Simulated data from the 2040–2069 scenarios were generated using the delta method (Croley 2003; Fagherazzi et al. 2005) and expressed as hydrologic series. Water flows from the Great Lakes watershed were simulated by Croley (2003) and took into account precipitation, evapotranspiration, ice cover, soil humidity, and surface water runoff. The water flows were then adjusted according to projected climate differences (monthly deltas), and new predictive hydrologic series were developed, incorporating the influence of climate change scenarios on St. Lawrence River flows.

A total of four hydrologic series were used in this study. The first two series were (1) the historically measured (MEAS) series, corresponding to the data observed between 1961 and 1989 (Morin and Bouchard 2000; Morin et al. 2003); and (2) the comparison (COMP) series, developed using the same method as for the climate change series, but taking into account previous climate data (1961–1989; Croley 2003; Fagherazzi et al. 2005; Morin et al. 2009). The COMP series was used to compare results with or without climate change, thereby identifying any biases associated with this comparison method. If the models were perfect, the COMP series would provide the same results as the MEAS series. The next two series, used for future climate conditions; were (3) the warm and wet (WAWE) series, associated with climate scenario HadCM3 A1FI; and (4) the warm and dry (WADR) series, associated with scenario CGCM2 A21. The MEAS and COMP series were roughly similar, but to fully understand the impact of climate change on hydrology, it was important to compare both climate change scenarios to the COMP series and not the MEAS series. Finally, to determine the impacts of climate change on wetlands, the model was initiated with reference conditions from 1960. Changes in the spatiotemporal distribution of the various classes of wetlands were determined by the hydrologic conditions associated with each hydrologic series (see Turgeon et al. 2005; Morin and Champoux 2006; and Morin et al. 2006, for details).

## Results

### Distribution of common reed stands

In 2010, common reed stands covered 212 ha in the entire study area, as revealed by the satellite images. Common reed covered 40 ha in Lake St. Louis, 86 ha in the Boucherville Islands (by far the most invaded sector), 34 ha in the Contrecœur Islands, and 52 ha in Lake St. Pierre, respectively. Mean common reed stand size was 14 m^2^ for the entire study area. In the Lake St. Pierre sector, it was only 3 m^2^, near the spatial resolution limit of pixels from the satellite images used. In Lake St. Pierre, 41% of common reed stands were small (≤10 m^2^), while in the other sectors, they were larger and in most cases ≥50 m^2^ (69% of reed stands in the Boucherville and Contrecœur Islands, and 57% in Lake St. Louis, respectively). Field mapping validation showed an increase in interpretation accuracy with common reed stand size. Small stands (≤10 m^2^) had a greater margin of error (21% were incorrectly identified) than medium (11–50 m^2^; 6%) and large (≥51 m^2^; 4%) stands. Overall, for all sectors and common reed stand sizes combined, the species identification at the stand level was correct in 88% of the cases. Reed canary grass and cattails (*Typha* spp.) were the other plant species involved in identification errors.

### Common reed model parameters and performance

The model which identified, on an annual basis, polygons from the IERM grid with potential for common reed seed germination and seedling survival, performed best using three parameters, that is, (1) temperature; (2) water level; and (3) wetland class. However, it is important to note that water level had a much greater influence on the model performance than the two other parameters combined. Removing these two parameters had virtually no impact on the model performance (0.05% decline in overall rate of correct classification). Nevertheless, because the three parameters collectively improved the model, they were all retained.

Comparing the actual distribution of common reed stands from 1980 to 2009 in the Boucherville Islands with the distribution of germination grounds produced using the model for the same period gave a correct (i.e., a stand occurred in a germination ground) classification rate of 57% (Table 1). The model sensitivity was 87%, while its specificity was only 49%. The best overall performance for the entire study area was in Lake St. Louis (65%). For all sectors, the model sensitivity clearly outperformed its specificity; for example, sensitivity reached 97% in the Contrecœur Islands, compared to 49% for specificity.

**Table 1:** Performance of the common reed (*Phragmites australis*) model that was developed to simulate the distribution of germination grounds of the species in wetlands in four sectors of the St. Lawrence River. Numbers are the proportion of polygons for which there is a match between the simulation of the model (presence of a germination ground) and the actual distribution of the common reed stands.

Sector	Sensitivity (%)	Specificity (%)	Rate of correct classification (%)
Lake St. Louis	82	65	65
Boucherville Islands	87	49	57
Contrecœur Islands	97	49	51
Lake St. Pierre	77	52	52

Approximately 62% of the polygons identified as germination grounds for the common reed in the Boucherville Islands, but containing no reed (according to the analysis of satellite images taken in 2010) and visited for validation purposes in 2012, contained at least one new reed stand. These new stands were generally small with only a few stems not connected to nearby stands; as a result, they were not detected on the 2010 satellite images, either due to their small size or because they were initiated in 2011 or 2012. The model performance increased considerably once these new small stands were included: The rate of correct classification for the validated subsector of the Boucherville Islands increased from 59% to 75%.

### Current and future distribution of common reed germination grounds

The grid used for modeling reduced the level of accuracy in assessing areas that were (or were likely to be) invaded, because common reed stands in the field were generally smaller than the polygons in which they were found. However, the model made no distinction between a polygon containing a small common reed stand and one completely covered. Therefore, when the model grid was applied to predict the current (2010) field situation, the surface area of common reed stands was inevitably overestimated. As such, due to the modeling scale used, common reed stands in 2010 were predicted to occupy 49 ha in Lake St. Louis (when in fact there were only 38 ha), 262 ha in the Boucherville Islands (vs. 86 ha), 100 ha in the Contrecœur Islands (vs. 34 ha), and 135 ha in Lake St. Pierre (vs. 52 ha), respectively.

**Figure 2:**
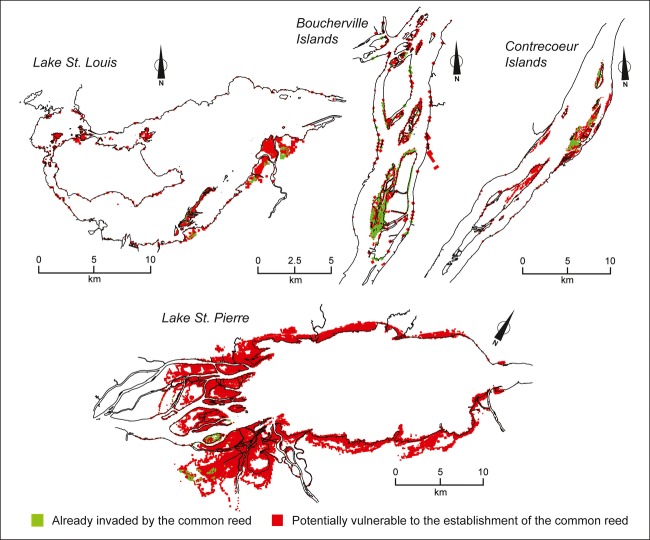
Spatial distribution of common reed (*Phragmites australis*) stands identified in four St. Lawrence River sectors by remote sensing images (2010) and modeled spatial distribution of germination grounds suitable for the species that have not yet been invaded. Germination grounds vulnerable to a common reed invasion are cumulated for the 1980–2009 period.

In all sectors, germination ground maps (Fig. 2) indicate that the common reed has probably not yet invaded all sites favorable to its establishment by seed. For example, in the Boucherville Islands, for the 1980–2009 period, 648 ha of wetlands having suitable conditions for the germination and survival of common reed seedlings was not yet invaded. In the other sectors and for the same period, surface areas not yet invaded totaled 1270 ha in Lake St. Louis, 1068 ha in Contrecœur Islands, and 13,118 ha in Lake St. Pierre, respectively. In other words, even though 29% (262 ha invaded out of a possible 910 ha) of suitable areas were already invaded in the Boucherville Islands, this proportion was much lower in Lake St. Louis (4%), the Contrecœur Islands (9%), and Lake St. Pierre (1%).

The method used for modeling the distribution of germination grounds of the common reed under future climate conditions also resulted in a loss of accuracy. For most years (86%), results produced with the COMP hydrologic series were less accurate than those from the MEAS series, all of which were obtained using quarter-monthly time steps. Differences between the COMP and MEAS series ranged from 3% to 35% for a given year; COMP values for total area of wetlands suitable for common reed germination and seedling survival were in almost all cases below MEAS values.

**Figure 3:**
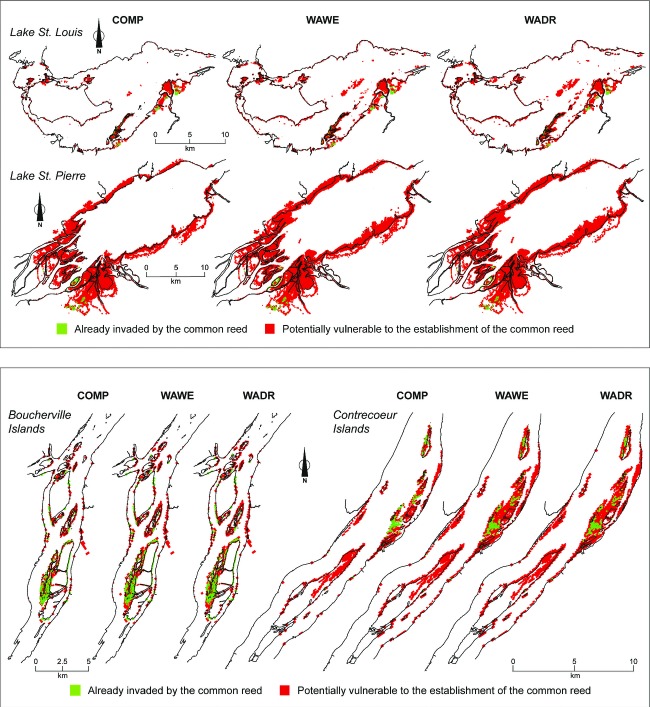
Spatial distribution of common reed (*Phragmites australis*) stands identified in four St. Lawrence River sectors by remote sensing images (2010) and modeled spatial distribution of germination grounds suitable for the species that have not yet been invaded, for three climate scenarios (COMP: the present-day climate 1961–1989; WAWE: warmer and wetter climate predicted for 2040–2069; WADR: warmer and drier climate predicted for 2040–2069). Germination grounds vulnerable to a common reed invasion are cumulated for the time period considered.

For the St. Lawrence River section between Lake St. Louis and Lake St. Pierre, hydrologic and temperature changes, associated with the two selected climate change scenarios, increased the surface area for the establishment of the common reed in wetlands (Table 2). Under climate conditions associated with the WAWE (wetter) and WADR (drier) scenarios, germination grounds covered 23% more surface area than those modeled with the COMP (reference) data (1961–1989). The difference was particularly high in Lake St. Louis (up to 50% increase) and in the Contrecœur Islands (up to 41%). There was little difference between the results of the WAWE scenario and those of the WADR scenario, except in Lake St. Louis, where the WADR scenario predicted greater surface areas. Under climate change conditions, sites conducive to common reed seed germination and seedling survival were found on a much larger portion of the floodplain and extended further from the bank toward the centre of the river (Fig. 3). In Lake St. Louis, new sites were present in the lake centre. In Lake St. Pierre, the formation of new common reed stands, extending from the lake banks to its centre, was especially notable.

**Table 2:** Total wetland area potentially suitable for the establishment of the common reed (*Phragmites australis*) through sexual reproduction in four sectors of the St. Lawrence River as estimated by an ecohydrological model and for three climate scenarios (COMP: present-day climate 1961–1989; WAWE: warmer and wetter climate predicted for 2040–2069; WADR: warmer and drier climate predicted for 2040–2069). The area potentially conducive to common reed establishment by seed is cumulated for the time period considered.

	Total wetland area suitable for common reed establishment (ha)				
Sector	COMP	WAWE	WADR	Difference WAWE versus COMP (%)	Difference WADR versus COMP (%)
Lake St. Louis	1486	1944	2228	+31	+50
Boucherville Islands	963	1113	1098	+16	+14
Contrecœur Islands	1014	1432	1400	+41	+38
Lake St. Pierre	13,935	16,876	16,660	+21	+20
All sectors	18,560	22,879	22,911	+23	+23

## Discussion

The common reed is not very abundant in the wetlands of the St. Lawrence River wetlands, but our model suggests that current climate conditions are already suitable for a considerable expansion of the species through sexual reproduction. Climate changes expected over the next 50 years may also exacerbate common reed expansion, particularly if combined with a water level drop, which will expose large bare areas suitable for seed germination. This expansion may be particularly noticeable in Lake St. Pierre, where existing common reed stands could increase their surface areas by a factor of 100, potentially creating the most extensive complex of reedbeds in North America, with over 13,000–16,000 ha. By comparison, the Delaware Bay is believed to currently have the largest reedbed complex in North America, covering a total of 2770 ha (Balletto et al. 2005). However, it is unlikely that all the common reed germination grounds predicted by the model will ultimately be invaded, because other invasive plants could also take advantage of low water levels to expand their populations, especially the reed canary grass and the flowering rush (*Butomus umbellatus* L.; see Lavoie et al. 2003, 2005; and Hudon 1997, 2004), which will compete with the common reed for space and resources (Vymazal and Krȍpfelová 2005). In the long term, however, even highly invasive species such as the reed canary grass will be outcompeted by the common reed (Březinová and Vymazal 2014).

The haplotype M is present along the St. Lawrence River for nearly 100 years, but has only covered to date a small portion of the area suitable for its establishment and growth. A biological invasion is often an exponential process: it remains relatively dormant over time before taking hold (lag phase; Crooks 2005; Pyšek and Hulme 2005; Aikio et al. 2010; Larkin 2012). A plant invader may take up to 150 years before reaching its maximum distribution (Gassó et al. 2010). Furthermore, the common reed is an opportunistic species, often establishing itself in wetlands following natural and anthropogenic disturbances (Chambers et al. 1999; Silliman and Bertness 2004; King et al. 2007; Hershner and Havens 2008). In the absence of disturbance, the existing vegetation provides some resistance to the invasion through competition for space and light, extending the lag phase (Lavoie et al. 2003; Byun et al. 2013). Differences in common reed stand size between the Lake St. Pierre sector and the other sectors of the St. Lawrence River may be due to fewer anthropogenic disturbances in Lake St. Pierre wetlands. The size difference may also be explained by the fact that the Lake St. Louis, Boucherville Islands, and Contrecœur Islands sectors are located in the heart of the most highly invaded region of southern Québec (Montérégie); the Lake St. Pierre area is located just outside this region (Jodoin et al. 2008). Consequently, the seed rain (propagule pressure; see Lockwood et al. 2005) is probably more important in the Montérégie region than in Lake St. Pierre.

Testing various combinations of parameters during the development of the model showed that a low water level at the beginning of the growing season is the best environmental predictor for the establishment of a new common reed stand through sexual reproduction. Including the type of wetland as additional parameter helped to improve the model performance, but this factor had much less influence than water level. Temperature has a definite impact on the duration of the growth season, but the interannual variability of areas with the potential for germination of the common reed seed (data not shown) was essentially tributary to variations in the river water level. Several studies showed that common reed seed germination occurs only in the presence of an exposed wetland substrate (Weisner and Ekstam 1993; Weisner et al. 1993; Coops and Van der Velde 1995; Baldwin et al. 2010). Other studies also showed that new common reed stands emerge close to large lakes and rivers, essentially during periods of low water levels (Whyte et al. 2008; Tulbure and Johnston 2010; Wilcox 2012).

One of the assumptions of the model was that the common reed shares roughly the same ecological niche in its native and exotic range. This is important, because most of the current knowledge regarding the biology of the common reed essentially comes from the native range (Europe). Niche conservatism is highly controversial for invasive species, and there is much conflicting evidence (for a review, see Guisan et al. 2014). In a recent paper, Guo et al. (2013) concluded that the niche of the exotic populations of the haplotype M of the common reed in eastern North America was shifted compared to the niche of the native populations. However, this study only compared temperature and precipitation envelopes associated with the distribution of the species. To what extent this conclusion also applies to germination and seedling survival requirements remains to be verified. We are nevertheless confident in our model given its good performance in the Boucherville Island sector. Indeed, the increased proportion of sites between 2010 and 2012 which fits model predictions, that is, sites identified by the model as suitable for seed germination and seedling survival and where reed stands were observed, confirms the accuracy of the model and the relevance of the selected parameters. In other words, failures of the model to correctly predict the presence or absence in the field probably reflect the early state of the invasion rather than a poor model performance.

Another model of common reed expansion in a context of future climate change has recently been published for the Great Lakes coastal zone (Carlson Mazur et al. 2014). Results are difficult to compare, because the scale and methods are completely different, but this model also predicts a substantial increase (from 3600 to 13,000 km^2^) by 2050 of the area suitable for the common reed, in a scenario where winters will become warmer and wetter. Water level changes were not specifically addressed, but the model suggests that common reed stands will likely expand in areas with low bathymetric slope, areas presumably corresponding to the shallowest sectors of the lakes. In both models, climate change is not a prerequisite for common reed expansion, but will probably accelerate the invasion, especially given that the species readily acclimatizes to rising temperatures and higher CO_2_ levels (Mozdzer and Megonigal 2012; Eller et al. 2013, 2014; Guo et al. 2013).

In summary, the model proposed in this article maps the sectors of the St. Lawrence River that are suitable for common reed establishment through sexual reproduction, under both current and future climates. It cannot, however, accurately predict when an invasion will occur or the speed at which it will progress. To do this, an algorithm for vegetative propagation would be necessary. It should also be noted that the climate change scenarios used in this article are among those presenting the greatest differences with the current climate (Morin et al. 2009). The resulting predictions are therefore particularly pessimistic. Notwithstanding, the common reed already has considerable room for expansion along the St. Lawrence River, regardless of climate change. Furthermore, hybridization (Paul et al. 2010; Saltonstall et al. 2014) may accelerate the invasion as hybrids are often more invasive than their parents in human-altered environments (Schierenbeck and Ellstrand 2009), and there are many populations of the native haplotypes of the common reed in Québec (Lelong et al. 2007). Negative impacts documented in brackishwater marshes of the coastal Atlantic regions of the United States (Meyerson et al. 2000; Dibble et al. 2013) presage the loss of biodiversity awaiting the biologically rich St. Lawrence River wetlands (Centre Saint-Laurent 1996) as the common reed continues to expand.
